# Should adrenaline be used in patients with hemodynamically stable anaphylaxis? Incident case control study nested within a retrospective cohort study

**DOI:** 10.1038/srep20168

**Published:** 2016-02-03

**Authors:** Byuk Sung Ko, Ji Yeon Kim, Dong-Woo Seo, Won Young Kim, Jae Ho Lee, Aziz Sheikh, David W. Bates

**Affiliations:** 1 Department of Emergency Medicine, University of Ulsan College of Medicine, Asan Medical Center, Seoul, Korea; 2 Department of Biomedical Informatics, Asan Medical Center, Seoul, Korea; 3 Allergy and Respiratory Research Group, Usher Institute of Population Health Sciences and Informatics, The University of Edinburgh, Edinburgh, UK; 4 Division of General Internal Medicine and Primary Care, Department of Medicine, Brigham and Women’s Hospital, Boston, Massachusetts, USA

## Abstract

Although adrenaline (epinephrine) is a cornerstone of initial anaphylaxis treatment, it is not often used. We sought to assess whether use of adrenaline in hemodynamically stable patients with anaphylaxis could prevent the development of hypotension. We conducted a retrospective cohort study of 761 adult patients with anaphylaxis presenting to the emergency department (ED) of a tertiary care hospital over a 10-year period. We divided the patients into two groups according to the occurrence of hypotension and compared demographic characteristics, clinical features, treatments and outcomes. Of the 340 patients with anaphylaxis who were normotensive at first presentation, 40 patients experienced hypotension during their ED stay. The ED stay of the hypotension group was significantly longer than that of patients who did not experience hypotension (496 min *vs* 253 min, *P* = 0.000). Adrenaline use in hemodynamically stable anaphylaxis patient was independently associated with a lower risk of developing in-hospital occurrence of hypotension: OR, 0.254 [95% CI, 0.091–0.706]. Adrenaline use in hemodynamically stable anaphylaxis patients was associated with a reduced risk of developing in-hospital occurrence of hypotension. Adverse events induced by adrenaline were rare when the intramuscular route was used.

Anaphylaxis is a serious, potentially fatal, systemic allergic reaction that develops rapidly after exposure to an offending agent^1^. The lifetime prevalence of anaphylaxis has been reported to be between 0.5–2%. The most common causes are drugs, foods and insect venom^2^,^3^,^4^,^5^. Recent studies have shown that the incidence of anaphylaxis is increasing in many countries^6^. Therefore, the prevention and treatment of anaphylaxis is important.

All major guidelines indicate that adrenaline (epinephrine) is the first-line recommended treatment in those experiencing anaphylaxis^7^,^8^,^9^,^10^,^11^. Delayed use of adrenaline has been shown to be associated with increased severity of reactions and fatalities^12^,^13^,^14^. However, various researches have consistently shown that adrenaline is under-used by physicians^15^,^16^,^17^. There are many reasons for this phenomenon. These include lack of physician’s knowledge about the presentation and recognition of anaphylaxis and fear of inducing adrenaline associated cardiovascular side-effects^18^,^19^,^20^,^21^. This happens more frequently particularly when patients initially present as normotensive because some practitioners still think that ‘shock’ needs to be present for a diagnosis of anaphylaxis^22^. Studies have however found that many cases of anaphylaxis do not manifest with cardiovascular shock; indeed, when it occurs anaphylactic shock is associated with particularly poor outcome and a high risk of fatality^23^,^24^,^25^.

We sought to investigate whether adrenaline use in hemodynamically stable patients can prevent the in-hospital occurrence of hypotension in hemodynamically stable patients with anaphylaxis.

## Results

**Figure 1:**
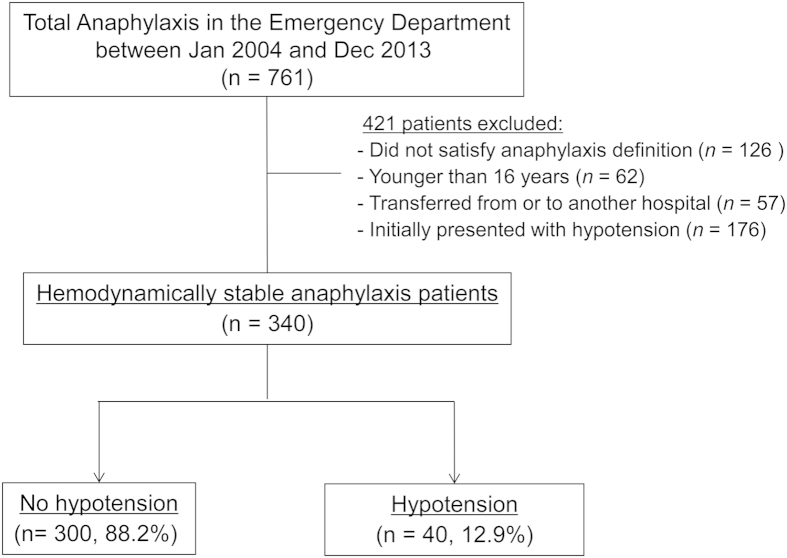
Patient flow diagram.

During the study period, 761 patients presented to the emergency department (ED) and were given a discharge diagnosis related to anaphylaxis. Of these, we excluded 126 patients whose diagnosis was not compatible with our pre-specified population and anaphylaxis definition criteria. The reasons for exclusion were: 176 patients with hypotension as an initial presentation at hospital after symptom onset, 62 patients younger than 16 years; 57 patients who were transferred from another hospital or to another hospital. We were thus left with a total of 340 hemodynamically stable (defined as systolic blood pressure ≥90 mmHg) patients with anaphylaxis in the final analysis (Fig. 1). The mean age was 45.6 ± 15.3 years and 52.1% were female. During their ED stay, 40 patients (11.8%) developed hypotension. The median time from first medical contact at hospital to the occurrence of hypotension was 35.0 (interquartile range (IQR) 9.0–116.0) minutes. The demographic characteristics, comorbidities, symptoms, signs and initial vital signs of the patients who developed hypotension versus those who did not are summarized in Table 1. Comorbidities, allergy history and anaphylaxis history were not significant different between the two groups. The initial systolic and diastolic blood pressures (BP) at first medical contact of patients who developed hypotension were significantly lower than those of patients who did not develop hypotension (114.1 vs 129.3 *P* = 0.000, 70.3 vs 81.1 *P* = 0.020, respectively).

No mortality was observed in ether group. The length of ED stay in the hypotension group was significantly longer than those of patients who did not experience hypotension (496 min vs 253 min, *P* < 0.001). In addition, a higher admission rate was observed in those experiencing hypotension when compared to those who remained normotensive (40% vs 15.7%, *P* = 0.001). Treatments between the two groups did not show any significant difference except for use of adrenaline in hemodynamically stable anaphylaxis patient (Table 2). Adrenaline use in hemodynamically stable anaphylaxis patient was less frequent in the hypotensive group (*P* = 0.010).

A stepwise logistic regression analysis with backward elimination was performed to identify independent variables that could predict hypotension development. Adrenaline use in hemodynamically stable anaphylaxis patient was associated with a reduced risk of developing hypotension (OR, 0.254 [95% CI, 0.091–0.706]) (Table 3).

There were two adrenaline induced adverse events. One patient was a 44 year old man, who presented with urticaria and dyspnea after taking ibupropen. He complained of chest discomfort after three minutes of receiving 0.1 mg adrenaline intravenous injection (whether dilution was performed or not was not confirmed) before development of hypotension. But it disappeared immediately after cessation of adrenaline and there were no abnormality on electrocardiogram and cardiac biomarkers. Another person was a 61 year old man with urticaria and hypotension. Adrenaline was administered after hypotension. Frequent ventricular premature beats were observed on his electrocardiogram after 0.1mg adrenaline intravenous injection (dilution was performed); he did not experience any symptoms. There was no cardiac biomarker elevation or any other adrenaline related adverse event.

## Discussion

Adrenaline use was associated with reduced risk of developing hypotension in patients experiencing anaphylaxis who were normotensive on presentation to hospital.

There were only two adrenaline induced adverse events, and neither resulted in permanent harm. Both were associated with the intravenous route and were thus used against guideline recommendations which clearly state that the intramuscular route is the preferred option. Clearly, adrenaline would have to be used with care in patients with underlying cardiac disease, but it appears that it is being underused in patients with anaphylaxis, and that higher rates of use has the potential to improve outcomes in this group.

To our knowledge, no study to date has reported association between use of adrenaline and the occurrence of hypotension in anaphylaxis. To date, adrenaline use for anaphylaxis has been largely based on expert opinion and relatively weak evidence; in particular, there have been no controlled trials and given the ethical and logistical challenge to mounting these in anaphylaxis these are unlikely to be forthcoming^21^,^22^. We have therefore we believe undertaken as rigorous a study as is possible at the present time.

Confounding by indication needs to be considered, although drug-induced anaphylaxis was more prevalent in the hypotension group, there was no difference in the severity between the two groups at initial presentation. That said, in the absence of undertaking a randomized controlled trial we cannot be sure that adrenaline prevented the development of hypotension. A randomized, controlled trial would be the best approach for addressing this issue. Although it has been reported that prophylactic use of adrenaline can substantially reduce the risk of anaphylaxis with anti-snake venom^26^, we did not find similar results with our study.

The primary outcome was defined as the development of hypotension during ED stay because mortality with anaphylaxis is mainly due to cardiovascular and respiratory compromise^14^. Hypotension reflects a severe generalized hypersensitivity reaction and is associated with poor outcomes^27^. Additionally, in our study, the hypotension group showed a longer ED stay and higher admission rate than the normotensive group due to stabilization of anaphylaxis and observation for more fatal reactions. Furthermore, the definition of respiratory compromise is difficult because there is no definite and objective parameter that reflects respiratory compromise in anaphylaxis. In contrast, cardiovascular compromise can be easily assessed by measurement of blood pressure and is relatively objective.

The main limitation of our present study was its retrospective study design. Accordingly, important information concerning clinical symptoms and past history of allergies or anaphylaxis and other factors may have been omitted. We could only trace the course and outcomes of patients in the ED, so there is a chance that the development of hypotension or significant biphasic reaction was missed. However, recent studies reported that clinically significant biphasic reactions in anaphylaxis are quite rare, so it is unlikely that mortality or a significant biphasic reaction including hypotension was underestimated^28^,^29^. Our study showed a small difference in triggers and clinical features compared with preexisting studies, so it might be difficult to generalize our results to all kinds of anaphylaxis situations. Further prospective studies are warranted.

Not all anaphylaxis symptoms and signs occur simultaneously. In some cases, anaphylaxis symptoms begin as a minor form, progressing so rapidly that no treatment can be given before respiratory or cardiac arrest^14^. Sampson *et al*.^23^ reported on fatal and near-fatal anaphylactic reactions to food in children and adolescents. Most of the patients presented skin and abdominal symptoms after 1 to 30 min that became severe between 20 min and 2.5 h later. The most important difference between fatal and nonfatal patients was that no patient in the fatal group received adrenaline before severe symptoms developed, whereas all patients with nonfatal reactions received adrenaline before or within 5 min of the development of severe symptoms. This clinical manifestation of anaphylaxis can be found in clinical practice. Thus, early administration of adrenaline should be considered, even if the first presentation is a mild form of anaphylaxis.

Adrenaline decreases mucosal edema, relieves upper airway obstruction, and increases blood pressure via an alpha-1 adrenergic vasoconstrictor effect. Beta-1 and -2 adrenergic receptor-mediated effects of adrenaline lead to inotropic and chronotropic effects and bronchodilation, which have beneficial effects in patients in anaphylaxis^20^,^21^. However, adrenaline also has adverse effects, from mild (i.e., pallor, tremor, anxiety, palpitations, headache, and dizziness) to severe (i.e., pulmonary edema, cardiomyopathy, left ventricular dysfunction, hypertension, cardiac arrhythmia, and myocardial infarction)^20^,^30^. These serious adverse events can occur by any route, but are most frequent after rapid intravenous infusion, an erroneous dose, or incorrect diluted adrenaline intravenous injection^8^,^30^. In our study, 2 of the 136 (all of adrenaline use before and after occurrence of hypotension) patients (1.5%) who received adrenaline experienced an adverse event, though neither resulted in long-lasting harm. Kanwar *et al*.^30^ reported a 2.4% incidence of potentially life-threatening complications from adrenaline. These complications were mainly from an inappropriate dose or route, but our two cases were not due to an incorrect dose, and the adverse events were not severe. Intramuscular, intravenous, and other routes comprised 82%, 13%, and 5% of all adrenaline administration in our study, respectively. All major guidelines indicate intramuscular adrenaline as a first line treatment of anaphylaxis because of safety problem compared to intravenous route. Despite 2 cases of adrenaline induced adverse event in our study were not life threatening, intramuscular route seems safer than intravenous route. As guidelines suggest, intravenous route should be saved for those who require repeated dose of intramuscular adrenaline or show refractory shock despite first line treatment.

In conclusion, adrenaline use in hemodynamically stable anaphylaxis patients was associated with reduced risk of in-hospital occurrence of hypotension. Intramuscular adrenaline should be used as a first line treatment of anaphylaxis because intravenous route can trigger potentially serious adverse events.

## Methods

### Study Design and Population

This incident case control study nested within a retrospective cohort study was conducted in the academic ED of a tertiary care, university-affiliated hospital in Seoul, Korea, that cares for approximately 110, 000 patients per year. Case was defined as the patients who experienced the development of hypotension during ED stay, and control was those who did not. Exposure of interest was adrenaline use in hemodynamically stable anaphylaxis patient, confounding factors were age, sex, history of anaphylaxis and treatment in ED. Due to the retrospective nature of the study, our institutional review board approved the review of patient data before its commencement and waived the requirement for informed consent. This study was carried out in accordance with the approved guidelines.

### Data Collection and Patient Management

The electronic medical records (EMRs) of all consecutive adult (≥16 years) patients with anaphylaxis who were hemodynamically stable (defined as systolic blood pressure ≥90 mmHg) at presentation in the ED or other hospital area (computed tomography or magnetic resonance imaging room and outpatient department) of our hospital between January 2004 and December 2013 were reviewed. Patient data were collected from a clinical data warehouse (CDW) which contains all EMR data. EMR system at ED was implemented January 2004. From the CDW, patients with discharge diagnosis of anaphylaxis related diseases (T780, T782, T805, T886 as ICD-10 Code) were selected and reviewed. Patients were included if they meet with diagnostic criteria for anaphylaxis according to the European Academy of Allergy and Clinical Immunology (EAACI)^1^,^31^. Two emergency physicians performed the review of medical charts together and reviewed the EAACI anaphylaxis guideline ahead of chart review. There were no uncertain cases for discussion.

Patients were excluded if they were younger than 16 years, did not satisfy the above definition of anaphylaxis or were transferred from or to other hospitals. We also excluded patients who already manifested hypotension on first presentation to the attending physician and/or nurse. We only used initial blood pressure checked in hospital and did not considered blood pressure checked in emergency medical service because such data were not stored in EMR database and sometime were not available even at time of ED arrival. The primary outcome was the development of hypotension, which was defined as systolic blood pressure <90 mmHg for more than 15 minutes without signs of other causes of shock except anaphylaxis progression during ED stay. Previous studies also used this primary outcome as severe form of anaphylaxis^32^,^33^. Our secondary outcome of interest was in-hospital mortality.

The clinical and demographic characteristics of all patients, including their age, sex, comorbidities, initial vital signs, laboratory findings, in-hospital course, treatment, and clinical outcomes, were retrieved from the CDW. The decision to perform treatment choice such as adrenaline, other adjunctive therapy was at the discretion of the treating physicians.

### Statistical Analysis

The data in this study were presented as the mean ± standard deviation or median with the interquartile range (IQR) for continuous variables and as absolute or relative frequencies for categorical variables. Patients who developed hypotension during ED stay were compared with those who did not. Student *t* tests or Mann-Whitney *U* tests were used to compare continuous variables, and chi-square tests were used for categorical variables. The results of logistic regression analysis of adrenaline use in hemodynamically stable anaphylaxis patient adjusted with age, sex and other significant factors in univariate analysis are presented as odds ratios (ORs) and 95% confidence intervals (CIs). A *p* value ≤ 0.05 was considered to be statistically significant. All statistical analyses were performed using SPSS for Windows version 18.0 (SPSS Inc. Chicago, IL, USA).

## Additional Information

**How to cite this article**: Ko, B. S. *et al*. Should adrenaline be used in patients with hemodynamically stable anaphylaxis? Incident case control study nested within a retrospective cohort study. *Sci. Rep*. **6**, 20168; doi: 10.1038/srep20168 (2016).

## Figures and Tables

**Table 1:** Baseline characteristics and clinical features of anaphylaxis patients according to the development of hypotension.

Variables	no hypotension (n = 300)	hypotension (n = 40)	*P* Value
Demographic factor			
Age, years	46.0 ± 15.5	42.7 ± 13.9	0.208
Male sex	145 (48.3)	18 (45.0)	0.738
Comorbidity			
Hypertension	39 (13.0)	5 (12.5)	1.000
Diabetes mellitus	11 (3.7)	0 (0.0)	0.374
Cardiac disease	14 (4.7)	3 (7.5)	0.705
Neoplasm	29 (9.7)	5 (12.5)	0.781
CKD	2 (0.7)	1 (2.5)	0.314
Bronchial asthma	13 (4.3)	1 (2.5)	1.000
History of allergy	109 (36.3)	13 (32.5)	0.727
History of anaphylaxis	25 (8.3)	3 (7.5)	1.000
Symptoms and Signs			
Mucocutaneous	261 (87.0)	34 (85.0)	0.803
Cardiovascular	110 (36.7)	17 (42.5)	0.490
Pulmonary	203 (67.7)	24 (60.0)	0.373
Gastrointestinal	93 (31.0)	19 (47.5)	0.048
Neurologic	63 (21.0)	21 (52.5)	0.000
Trigger			
Drug	75 (25.0)	20 (50.0)	0.001
Contrast media	48 (16.0)	3 (7.5)	0.236
Food	106 (35.3)	10 (25.0)	0.218
Insect venom	18 (6.0)	2 (5.0)	1.000
Contact	4 (1.3)	1 (2.5)	0.467
Idiopathic	47 (15.7)	4 (10.0)	0.480
Severe on severity Grade^a^	48 (16.0)	9 (22.5)	0.365
Initial vital signs			
Systolic BP, mmHg	129.3 ± 23.4	114.1 ± 24.7	0.000
Diastolic BP, mmHg	81.1 ± 17.3	70.3 ± 27.7	0.020
Heart rate, beats/min	90.9 ± 21.4	88.8 ± 23.2	0.562
Respiratory rate, breaths/min	21.6 ± 3.6	22.9 ± 5.7	0.170
Oxygen saturation, %	97.5 ± 2.6	96.3 ± 3.6	0.052

CKD: chronic kidney disease; BP: blood presssure.

Values are expressed as mean ± SD, n (%).

^a^Severe was defined as cyanosis or SpO^2^ ≤ 92%, hypotension (systolic BP < 90 mm Hg), confusion, collapse, loss of consciousness, or incontinence at admission.

**Table 2:** Treatments and outcomes of anaphylaxis patients according to the development of hypotension.

Variables	no hypotension (n = 300)	hypotension (n = 40)	*P* Value
Treatment			
H1-antihistamines	269 (89.7)	37 (92.5)	0.781
H2-antihistamines	255 (85.3)	37 (92.5)	0.239
Corticosteroids	243 (81.0)	35 (87.5)	0.389
Auto injector	6 (2.0)	0 (0.0)	1.000
Salbutamol nebulizer	55 (18.3)	5 (12.5)	0.393
Use of adrenaline in hemodynamically stable	101 (33.7)	5 (12.5)	0.010
Admission	47 (15.7)	16 (40.0)	0.001
ED stay, minutes	253 (152–404)	496 (246–821)	0.000
Medical contact to hypotension		35.0 (9.0–116.0)	NA

ED: emergency department; NA: nonapplicable.

Values are expressed as median and interquartile range (IQR), or n (%).

**Table 3:** Factors associated with the development of hypotension.

Variables	OR	*P* value	95% CI
Use of adrenaline in hemodynamically stable	0.254	0.009	0.091–0.706

CI: confidence interval; OR: odds ratio.
